# The Investigation of Nutritional Status, Intestinal Permeability, and Quality of Life in People with Celiac Disease

**DOI:** 10.5152/tjg.2022.21815

**Published:** 2022-12-01

**Authors:** Yeşim Öztekin, Fatma Esra Güneş, Yeşim Özen Alahdab

**Affiliations:** 1 Department of Nutrition and Dietetics, Hacettepe University, Ankara, Turkey; 2 Department of Nutrition and Dietetics, Marmara University, İstanbul, Turkey; 3 Department of Gastroenterology, Marmara University, İstanbul, Turkey

**Keywords:** Celiac disease, gluten-free diet, intestinal permeability, quality of life, zonulin

## Abstract

**Background:**

Celiac disease is associated with impaired intestinal epithelial barrier integrity. Its consequences affect the nutritional status and quality of life of patients. This study aimed to determine nutritional status, intestinal permeability, and quality of life in people with celiac disease. It was researched whether patients who are non-compliant to gluten-free diet have higher serum zonulin levels and intestinal permeability.

**Methods:**

The study was completed with 44 celiac patients. Dietary records and a questionnaire were used to evaluate patients’ compliance to the gluten-free diet. Dietary records were analyzed by using a nutrition analysis program. Anthropometric measurements were taken. Body compositions were analyzed to assess the nutritional status of patients. Blood samples were collected and then zonulin levels and total serum proteins were measured to evaluate intestinal permeability. Celiac Disease Questionnaire was used to assess the quality of life scores.

**Results:**

Patients were divided into 2 groups considering compliance to the gluten-free diet and it was found that 17 patients were compliant to the gluten-free diet, and 27 patients were non-compliant to the gluten-free diet. Serum zonulin, zonulin/total protein ratio, and intestinal permeability were higher in non-compliant to the gluten-free diet group (*P* < .05). There was no significant difference between anthropometric measurements, Celiac Disease Questionnaire scores, daily energy, and nutrient intakes of groups (*P* > .05).

**Conclusion:**

The content of a gluten-free diet has a decisive role in the nutritional status and quality of life of celiac patients. Additionally, zonulin levels and intestinal permeability were higher in people with celiac disease who are non-compliant to gluten-free diet.

**Main Points**

- Compliance to a gluten-free diet decreases intestinal permeability in people with celiac disease.

- Zonulin levels are lower in people with celiac disease who are on a gluten-free diet.
- The nutritional quality of a gluten-free diet affects the nutritional status and anthropometric measurements of celiac patients.
- The first effect of compliance to a gluten-free diet in terms of quality of life can be seen as an increase in gastrointestinal health-related quality of life.

## Introduction

Celiac disease (CD) is defined as an autoimmune enteropathy that progresses with malabsorption of gluten protein found in wheat, barley, and rye. It is characterized by mucosal inflammation of the small intestine in individuals with a genetic predisposition.^1^ In recent studies, it has been stated that the intestinal epithelial membrane is impaired in celiac patients. The epithelial membrane acts as a barrier between the intestinal lumen and lamina propria so it is called as an intestinal barrier.^2^ The most important structures that ensure the integrity of the intestinal barrier are tight junction proteins. Exposure to gluten damages tight junctions and disrupts the intestinal barrier. This situation results in paracellular passages from the intestinal lumen to lamina propria and increases intestinal permeability.^3^ Zonulin is a pre-haptoglobin and modulator of tight junctions. Studies showed that indigestible gluten peptides bind more to chemokine receptors and release zonulin in people with CD. Increased zonulin concentration in blood is considered an indicator of increased intestinal permeability.^4^ Currently, the only treatment for CD is a lifelong gluten-free diet (GFD). A decrease in villous atrophy, intestinal permeability, and nutrient malabsorption was observed with a GFD.^5,6^ While compliance to the GFD is a crucial point to prevent the progression of the disease, the nutritional quality of the GFD affects the nutritional status and quality of life in patients. Gluten-free products generally have lower fiber, iron, pyridoxine, riboflavin, thiamin, niacin, and folate compared to standard gluten-containing products.^7^ Therefore, an adequate and balanced GFD plays an essential role in the nutritional status of celiac patients. Moreover, symptoms of the disease and nutritional therapy with gluten-restricted diets influence health-related quality of life. In order to determine the quality of life of celiac patients, Celiac Disease Questionnaire (CDQ) was developed.^8^ The Turkish version of CDQ was validated for people with CD living in Turkey.^9^ Accordingly, the purpose of the study is to investigate intestinal permeability by measuring serum zonulin levels in celiac patients. In addition, the study aimed to evaluate the nutritional status and determine the quality of life of people with CD.

## Materials and Methods

Ethics committee approval was received from the Marmara University (approval no: 09.2018.359/acceptance date: May 04, 2018). The study was conducted in accordance with the ethical principles of the 1964 Declaration of Helsinki and its later amendments. Adult celiac patients who applied to the gastroenterology unit in a hospital between May 2019 and October 2019 were accepted as the population of the study. Inclusion criteria for the study were given as follows: patients should be between the ages of 19 and 65, should have a small intestinal biopsy-proven CD diagnosis, and should be a volunteer by signing the informed consent form. Patients who have another autoimmune disease such as type 1 diabetes, psoriasis, Hashimoto’s thyroid, rheumatoid arthritis, systemic lupus erythematosus and who are in pregnancy or lactation period were excluded. Patients who were taking probiotic/prebiotic supplements in the last 2 months and taking antibiotics or non-steroidal anti-inflammatory drugs in the past week were also excluded. All patients who met the inclusion and exclusion criteria and provided informed consent were included in the study.

In previous studies, it was emphasized that dietary records and nutritional history taken by a dietitian were the best methods for the evaluation of compliance to a GFD.^10,11^ In this consideration, an experienced dietitian interviewed each patient for 30-45 minutes. During the dietitian interview, 3-day dietary records and nutritional history of patients were taken. In nutritional history, 7 open-ended questions were asked to evaluate compliance with the GFD. With these questions, the evaluation of patients’ knowledge level about CD and gluten-free nutrition was aimed. The questions for the evaluation of compliance to GFD were given in Table 1. In food records, patients were asked to state the brands of the packaged foods they consume. Therefore, it can be understood whether the diet is exactly gluten-free or not. Considering the nutritional history and dietary records of patients, they were divided into 2 groups as compliant to the GFD and non-compliant to the GFD (NGFD) group.

### Measurement of Serum Zonulin Levels

Venous blood samples taken from the participants after 8-12 hours of fasting should be used in the zonulin test. Each blood sample was centrifuged for 15 minutes at 1500 rpm at +4°C (Allegra X-I5R; Beckman Coulter Inc., USA), and 1 mL of serum sample was separated and stored at −80°C in Eppendorf tubes until zonulin analysis. The assays are based on the sandwich enzyme-linked immunosorbent assay (ELISA) method, and human zonulin ELISA kits (Cusabio) were used. All assays were performed according to the manufacturer’s kit protocol, and the tests were duplicated for each sample. 100 µL of each blood sample was added to each well of the plate and incubated. The liquid in the wells was removed and 100 µL of biotin-bound antibody was added to each well. After incubation, the liquid in the wells was removed, and each well was washed with distilled water. Then, 100 μL of Horseradish Peroxidase (HRP)-avidin enzyme was added and incubated at 37°C for 1 hour. The liquid was removed and washed 5 times. 90 µL of tetramethylbenzidine substrate was added and incubated, and the reaction was terminated by adding 50 µL of stopping solution to each well. Absorbance values were read in 450 nm spectrophotometer within 5 minutes. Zonulin concentrations were calculated in milliliters of total protein in nanograms (ng/mL). In addition, the total protein amount of serum samples was measured in milligrams (mg/mL) per milliliter by using bicinchoninic acid kit (Pierce; Thermo Fisher Scientific Inc.; Massachusetts, USA). Thus, the ratio of zonulin amount to total protein amount in serum was calculated (ng/mg).

### Determination of Nutritional Status

A 3-day dietary record was maintained for 3 non-consecutive days. All foods and drinks, their contents, and brand names were recorded. Food records were analyzed in the Nutrition Information Systems (BEBIS) program. The mean value of energy and nutrient intake was calculated for each individual. These values were compared with reference values published in Dietary Guidelines for Turkey (TUBER) in 2015.^12^ Anthropometric measurements of each individual were taken. Height was measured with a stadiometer. Weight (kg) and body composition components were measured with a bioelectrical impedance analysis-based body analyzer (Inbody 120; Inbody, South Korea). Measurements of body composition were determined by following both standard protocols and manufacturer’s guidelines. Height and body composition were measured with bare feet. In order to increase the accuracy of measurement, participants were requested to fast overnight (8-12 hours) and to remove metallic accessories before body composition analysis. Body mass index (BMI) was calculated as weight divided by squared height (kg/m^2^). Body mass index values were categorized according to the World Health Organization criteria as follows: <18.5 kg/m^2^ as underweight, 18.5-24.9 kg/m^2^ as normal weight, 25-29.9 kg/m^2^ as overweight, and ≥30 kg/m^2^ as obese.^13^ Waist and hip circumference were measured, and the ratio of waist circumference to hip circumference was calculated.

### Determination of Celiac Disease Questionnaire Scores

Celiac Disease Questionnaire was performed on all participants by face-to-face interview method. The questionnaire has 4 subscales for the emotional, social, gastrointestinal, and anxiety states of the patients. Celiac Disease Questionnaire consists of 28 questions, and each question was evaluated with 7-point Likert scale so that the total score is between 0 and 196 points and subscale scores are between 0 and 49 points. The low score indicates the low health-related quality of life and the high score indicates the high health-related quality of life for celiac patients. Patients were given the opportunity to not answer a question if they do not want to.

### Statistical Analysis

Statistical Package for the Social Sciences version 24.0 program (IBM Corp.; Armonk, NY, USA) was used to analyze the data. Normality of distribution was tested using the Shapiro–Wilk test. The Mann–Whitney *U* non-parametric test was performed for data in the non-normal distribution, and Student’s *t*-test was performed for data in the normal distribution. The chi-Square test was used for categorical variables. Pearson and Spearman’s tests were performed for correlation analysis. In all analyses, *P* < .05 was accepted as statistically significant.

## Results

**Figure 1.**
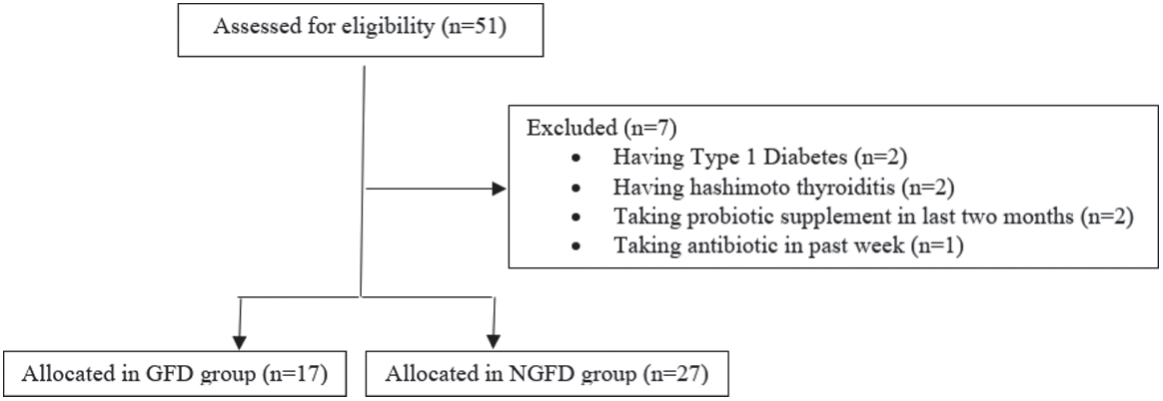
Flowchart of inclusion of participants in the current study. GFD, compliant to gluten-free diet group; NGFD, non-compliant to gluten-free diet group.

The study was completed with 44 celiac patients who met the inclusion and exclusion criteria. The gluten-free diet group consists of 17 celiac patients, and the NGFD group consists of 27 patients. The allocation of participants was shown in Figure 1. The mean age of the NGFD group was 36.23 ± 14.07 years, while the GFD group was 36.37 ± 11.33 years. Table 2 indicates the clinical and demographic characteristics of the groups. There was no significant difference between the groups in terms of clinical and demographic characteristics (*P* > .05). Serum zonulin levels, total protein amount, and serum zonulin levels to total protein ratio were given in Table 3. Serum zonulin level and zonulin/total protein ratio were significantly higher in the NGFD group compared to the GFD group (*P* < .05).

There was no statistically significant difference between groups in terms of energy and macronutrient intake levels (*P* > .05). In both the groups, energy and carbohydrate and dietary fiber intake were lower than TUBER reference values, while protein and fat intake was higher than the reference values. There was no significant difference between the groups in terms of certain micronutrient intakes (*P* > .05). Intake of thiamine, riboflavin, folate, vitamin B_6 _and B_12__,_ zinc, iron, calcium, and magnesium was lower than the reference values in both groups. The comparison of participants’ energy and nutrient intake was given in Table 4. Data on the anthropometric measurements of the groups were shown in Table 5. Body mass index, waist circumference, and waist/hip ratio were higher in NGFD group. However, there was no significant difference between all anthropometric measurements of GFD and NGFD groups. Table 6 includes mean and median values of total CDQ score and subscale scores of groups. When the total CDQ score and all subscale scores were compared between the groups, no significant difference was found (*P* > .05).

## Discussion

In the present study, significantly higher zonulin levels were found in celiac patients in the NGFD group than GFD group. However, anthropometric measurements and energy and nutrient intake and quality of life scores of groups were similar.

It was known that CD is characterized by intestinal epithelial barrier disruption and nutritional malabsorption. Intestinal permeability is one of the intestinal anatomical disorders caused by gluten intake.^7,8^ Higher zonulin levels were found in people with CD compared to healthy people.^14^ However, intestinal permeability is reversible with compliance to GFD. Duerksen et al^15^ investigated the relationship between GFD compliance and zonulin levels in people with CD and observed that the group consuming a gluten-containing diet has the highest zonulin levels. The emphasized point in the study is the decreased zonulin levels and intestinal permeability with compliance to a GFD. Although GFD is currently the only treatment for CD, pharmacological researches have been conducted. Larazotide acetate is a zonulin inhibitor and is a promising pharmacological treatment option for CD. The administration of larazotide acetate reduced the symptoms of celiac patients.^16^ These treatment strategies on zonulin and intestinal permeability strengthen the relationship between increased intestinal permeability and CD. Increased intestinal permeability exacerbates the malabsorption of nutrients.^17^ Hence, previous studies emphasized the importance of adherence to a GFD.^17,18^ On the other hand, this study shows that energy and nutrient deficiencies can be seen in people with CD even if they adhere GFD. A study conducted in Turkey showed that the daily energy intake was lower in celiac patients than in healthy people.^19^ Insufficient energy intake also can be a sign of poor food intake. Similarly, it was found in our study that the energy and carbohydrate and dietary fiber intake of both GFD and NGFD groups were lower than the reference values. It has been stated in various studies that the intake of dietary fiber is inadequate in people with CD.^20,21^ One of the reasons for inadequate fiber intake is the low fiber in gluten-free products because gluten-free products generally contain potato, corn, and rice starch.^22^ Micronutrient composition such as iron, pyridoxine, riboflavin, thiamin, niacin, folate, manganese, and vitamin B_5_ of gluten-free products analyzed by Larretxi et al^7^ was lower in gluten-free products as compared to standard gluten-containing products. In a systematic review and meta-analysis study published in 2019, insufficient iron, zinc, vitamin D, B_12_, and folate levels were found in celiac patients even if patients adhere to a GFD.^23^ Therefore, the composition of GFD has a pivotal role in the nutritional status of patients. The mean value of BMI in both GFD and NGFD groups was found in the normal range in our study. However, Ukkola et al^24^ observed low BMI in adult patients who do not adhere to a GFD. On the other hand, changes can be seen in the nutritional status of patients. A study published by Kabbani et al^25^ showed that after adherence to a GFD, the BMI value shifted from underweight class to normal or overweight class in 15.8% of celiac patients. Excessive consumption of gluten-free hypercaloric packaged products by celiac patients can be a reason for the increase in BMI. Moreover, compliance to a GFD decreases malabsorption and increases the bioavailability of nutrients in the body.

Celiac patients who cannot adhere to a GFD have a lower health-related quality of life.^26^ In the present study, there is no significant difference between the quality of life scores of GFD and NGFD groups. However, the highest quality of life score was seen in the gastrointestinal symptom subscale of the GFD group. This situation supports the positive effects of GFDs on gastrointestinal symptoms. Additionally, higher gastrointestinal and emotional subscale scores and lower social and anxiety subscale scores were seen in the GFD group. These subscale scores can be affected by life conditions. Therefore, the impact of a GFD on the quality of life may not be seen.

Similarly, Deepak et al^27^ revealed that gastrointestinal symptoms decreased as the first effect of a GFD when celiac patients began to consume a GFD. The result of a systematic review and meta-analysis study indicated that a GFD increased the health-related quality of life scores of patients.^28^ A limitation of our study is that recall and self-report problems of dietary records. Additionally, the study was a single-center trial with small sample size.

In conclusion, adherence to a GFD is still challenging for people with CD. A considerable number of patients consume gluten in their diet. However, intake of gluten increases zonulin levels and intestinal permeability. It should be noted that a GFD is the only treatment for CD. This study indicated whether intestinal disruption and permeability can be reversed in people with CD who adhere to a GFD. Moreover, the nutritional composition of the GFD should be adequate and balanced to prevent nutrient deficiencies. With compliance to a nutritious GFD, improvement in intestinal permeability, nutritional status, and quality of life can be observed in people with CD.

## Figures and Tables

**Table 1.** Questions for the Evaluation of Compliance to Gluten-free Diet

1 What are celiac disease and gluten? Which foods contain gluten?
2 How did you learn about the gluten-free diet after the diagnosis?
3 What is your eating frequency in cafes or restaurants?
4 Are there any gluten-containing foods you have consumed in the past 4 weeks? And what is it?
5 What is cross-contamination? What are the precautions that you take against cross-contamination of gluten?
6 What do you pay attention to the label of a product when buying packaged products?
7 What is the meaning of the “it may contain trace amount gluten” expression?

**Table 2.** Demographic and Clinical Characteristics

GFD Group	NGFD Group				***P***
	**n**	**%**	**n**	**%**	
**Gender **					.218
Female	5	29.4	13	48.1	
Male	12	70.6	14	51.9	
**Educational status**					.692
Primary school	1	5.9	5	18.5	
Secondary school	2	11.8	5	18.5	
High-school	5	29.4	8	29.6	
University	9	53.0	9	33.3	
**Working status**					.579
Employed	10	58.9	14	51.9	
Unemployed	7	41.1	13	48.1	
**Smoking status**					.090
Yes	2	11.8	10	37	
No	15	88.2	17	63	
**Compliance to gluten-free diet**					.008
None of the time	0	0	3	11.1	
Hardly any of time	0	0	0	0	
Some of the time	2	11.8	6	22.2	
Most of the time	15	88.2	7	25.9	
All of the time	0	0	11	40.7	
**The diagnosis of other disease**					.688
Yes	4	23.5	5	18.5	
No	13	76.5	22	81.5	
**Use of medication**					.137
Yes	1	5.9	6	22.2	
No	16	94.1	21	77.8	

GFD, compliant to gluten-free diet group;

NGFD, non-compliant to gluten-free diet group.

**Table 3.** Serum zonulin, Total Protein Levels, and Zonulin/Total Protein Ratio of Groups

	**GFD Group Mean ± SD**	**Median**	**NGFD Group Mean ± SD**	**Median**	***P***
Serum zonulin levels (ng/mL)	2.31 ± 1.16	2.42	4.55 ± 2.61	3.77	.001*
Total protein (mg/mL)	4.56 ± 0.049	4.60	4.59 ± 0.036	4.60	.470
Zonulin/total protein (ng/mg)	0.50 ± 0.25	0.53	0.97 ± 0.59	0.82	.002*

Data were expressed as mean ± standard deviation (SD) and median value.

GFD, compliant to gluten-free diet group;

NGFD, non-compliant to gluten-free diet group.

*P < .05.

**Table 4.** Daily Energy and Nutrient Intake of Groups

	**GFD Group ** **Mean ± SD**	**Median**	**NGFD Group ** **Mean ± SD**	**Median**	TUBER* Reference Values	***P***
Energy (kcal)	1408.63 ± 342.77	1378.6	1378.86 ± 423.56	1339.54	1800-2200	.656
**Macronutrients**						
Carbohydrate (TE %)	43.76 ± 9.3	41	44.11 ± 8.85	45	50-60	.546
Protein (TE %)	16.64 ± 4.79	15	16.22 ± 3.16	18.72	15-20	.952
Fat (TE %)	39.52 ± 6.84	38.81	38.81 ± 6.28	41	20-35	.735
Dietary fiber (g)	14.51± 5.58	13.5	13.44 ± 6.60	11.76	>25	.419
**Micronutrients**						
**Vitamins**						
Thiamine (mg)	0.64 ± 0.23	0.61	0.60 ± 0.28	0.55	1.1-1.2	.477
Riboflavin (mg)	0.96 ± 0.26	1.01	1.1 ± 0.63	0.92	1.1-1.3	.800
Vitamin B_6_ (mg)	1.02 ± 0.4	1.1	1.11 ±0.63	0.95	1.3-1.7	.987
Folate (mg)	217.06 ± 79.58	207.12	231.3 ± 105.7	215.57	330	.876
Vitamin B_12 _(μg)	3.68 ± 21.16	3.22	6.15 ± 8.92	3.04	4	.923
**Minerals**						
Zinc (mg)	8.2 ± 2.5	7.96	7.91 ± 3.36	7.37	7.5-16.3	.433
Iron (mg)	8.79 ± 2.41	8.03	8.25 ± 3.3	8.05	11-16	.515
Calcium (mg)	584.64 ± 215.17	215.16	541.37 ± 218.19	208.19	950-1000	.477
Magnesium (mg)	219.31 ± 81.07	210.43	183.47 ± 77.83	165.10	300-350	.120

Data were expressed as mean ± standard deviation (SD) and median value.

GFD, compliant to gluten-free diet group; NGFD, non-compliant to gluten-free diet group; TE%, percentage of total energy.

*TUBER: Dietary Guidelines for Turkey.

**Table 5.** Anthropometric Measurements of Groups

Anthropometric Measurements	GFD Group		NGFD Group		*P*
	Mean ± SD	Median	Mean ± SD	Median	
**Body weight kg)**	65.74 ± 16.4	61.4	69.49 ± 15.16	67.9	.233
**Height (cm)**	167.82 ± 9.83	167	168.33 ± 8.16	169	.791
**BMI (kg/m ^2^ )**	23.07 ± 3.28	22.8	24.42 ± 4.60	23.9	.341
**Waist circumference (cm)**	84.05 ±13.04	82	89 ± 12.65	89	.138
**Hip circumference (cm)**	97.23 ± 6.54	97	100.11 ± 9.41	100	.353
**Waist/hip ratio**	0.86 ± 0.09	0.87	0.88 ± 0.07	0.90	.201
**Fat mass (%)**	25.64 ± 7.32	24.5	24.87 ± 10.79	25.6	.772
**Muscle mass (kg)**	27.54 ± 7.88	25.00	29.18 ± 6.57	28.10	.189

Data were expressed as mean ± standard deviation (SD) and median value.

GFD, compliant to gluten-free diet group; NGFD, noncompliant to gluten-free diet group; BMI, body mass index.

**Table 6.** CDQ Scores of Groups

Total Scores and Subscale Scores	GFD Group Mean ± SD	Median	NGFD Group Mean ± SD	Median	*P*
**Total **	132.18 ± 21.78	133	130.56 ± 24.74	139	.962
**Emotional **	31.53 ± 6.36	33	29.59 ± 8.71	32	.425
**Social**	34.18 ± 6.74	32	35.11 ± 7.55	35	.638
**Anxiety **	29.89 ± 7.73	33	30.92 ± 9.03	31	.772
**Gastrointestinal **	38.35 ± 6.40	39	35.74 ± 7.49	35	.954

Data were expressed as mean± standard deviation (SD) and median value.

GFD, compliant to gluten-free diet group;

NGFD, non-compliant to gluten-free diet group.
